# Yerba Maté (Illex Paraguariensis) ingestion augments fat oxidation and energy expenditure during exercise at various submaximal intensities

**DOI:** 10.1186/1743-7075-11-42

**Published:** 2014-09-02

**Authors:** Ahmad Alkhatib

**Affiliations:** 1 Academy of Sport and Physical Activity, Faculty of Health and Wellbeing, Sheffield Hallam University, Sheffield, S10 2BP, UK; 2 Sport Science Program, College of Arts and Sciences, Qatar University, Doha, P.O. Box 2713, Qatar

**Keywords:** Plant, Ingestion, Metabolism, Weight loss, Thermogenic

## Abstract

**Background:**

Ingesting Yerba Maté (YM) has become widely popular for health promotion, obesity prevention and body weight reduction, primarily due its thermogenic effectiveness. However, the YM effects on fat metabolism during exercise, when fat metabolism is already increased several fold, are unknown. The present study investigated whether acute YM ingestion augments fat metabolism parameters of fatty acid oxidation (FAO) and energy expenditure derived from FAO (EE_FAO_) during exercise with several intensities.

**Methods:**

Fourteen healthy males and females were randomised in a repeated measures crossover experimental design. All participants ingested either 1000 mg of YM or placebo capsules (PLC) 60 min before performing two incremental exercise ergometry tests. Power output was initiated at and increased by 0.5 W.kg^-1^ of body weight every 3 min stage, until reaching peak oxygen uptake $\left(\dot{V}O_{2_{\;\mathrm{Peak}}}\right)$. Expired gases and stoichiometric indirect calorimetry were used to analyse FAO and EE_FAO_. Capillary blood samples were collected and analysed for blood lactate concentration (BLC) at rest and at each submaximal and maximal power output.

**Results:**

YM significantly increased FAO and EE_FAO_ by 24% in all submaximal exercise intensities below 70% of $\dot{V}O_{{2\;}_{\mathrm{peak}}}$ (p < 0.001, ANOVA main effects) with post hoc tests showing a higher FAO and EE_FAO_ (p < 0.05) at the lower exercise intensities (e.g. 0.26 ± 0.09 vs. 0.35 ± 0.10 and 0.25 ± 0.12 vs. 0.33 ± 0.11 g.min^-1^ at 40 and 50% of $\dot{V}O_{{2\;}_{\mathrm{peak}}}$ respectively). These changes were combined with a trend towards a decrease in BLC (*P* = 0.066), and without a significant difference in $\dot{V}O_{{2\;}_{\mathrm{peak}}}$, peak power, peak RER, or peak BLC.

**Conclusions:**

Acute YM ingestion augments the exercise dependent increase in FAO and EE_FAO_ at submaximal exercise intensities without negatively affecting maximal exercise performance, suggesting a potential role for YM ingestion to increase the exercise effectiveness for weight loss and sports performance.

## Introduction

Yerba Maté (YM), the dried leaves and branches of the plant Illex Paraguariensis, is currently consumed by over 1 million people worldwide, traditionally by many South American countries, and more recently in North America and Europe in the form of YM tea beverage made from the aqueous extracts of the dried leaves and stem. The active ingredients of YM include polyphenols and caffeoyl derivatives (caffeic acid, chlorogenic acid, 3, 4-Dicaffeoylquinic acid, 4, 5-Dicaffeoylquinic acid and 3,5-Dicaffeoylquinic acid), phytosterols and saponins [1,2]. These active ingredients have been suggested to explain several biomedical properties associated with YM ingestion including anti-oxidant, vasodilatory, lipid lowering properties, anti-mutagenic and anti-glycation effects [3]. These properties have often accompanied weight and fat loss and increased energy metabolism as recently demonstrated in mice studies [4-7].

The reported effects in humans are limited to one study that showed an increase in resting metabolic rate and reduced respiratory quotient after prolonged resting periods of 1–4 hrs, induced by acute YM ingestion [8]. However, those resting effects may be further augmented during exercise, especially considering that energy metabolism is already stimulated as a result of increasing exercise intensity. It is established that fatty acid oxidation (FAO) predominantly contributes to energy expenditure (EE) at low and moderate exercise intensities and that carbohydrate oxidation (CHO) predominates at higher exercise intensities above anaerobic threshold [9,10]. It has been shown that achieving higher absolute FAO and higher EE derived from FAO (EE_FAO_) at a given exercise intensity or power output, particularly those associated with aerobic exercise training in the low and moderate intensity domains, is associated with improved metabolic health and enhanced endurance exercise performance outcomes [11-15]. Therefore, given the suggested thermogenic effectiveness of YM at rest [8], it remains unknown whether and how YM ingestion affects FAO and its contribution to EE during exercise.

This study aims to investigate the acute effects of YM ingestion on FAO and EE derived from FAO (EE_FAO_) during exercise of varied intensities. It is hypothesised that YM ingestion increases FAO and enhances EE_FAO_ during exercise.

## Methods

### Design and participants

The study followed a double-blind crossover repeated measures experimental design. All environmental conditions were maintained the same during all experimental conditions (Mean ± SD: 20 ± 1°C, 775.6 ± 12 mmHg and 51.1 ± 6.1%) for air temperature, barometric pressure and relative humidity respectively. The study gained institutional ethical approval and was carried out in accordance with the Declaration of Helsinki of the World Medical Association and all participants gave their written informed consent to participate. Sample size calculations were based on achieving a large effect size based on the least meaningful difference induced by thermogenic supplements on EE in previous studies [16], and provided a power of 90% for a significance alpha level of 5%.

The participants were fourteen healthy adults, seven males and seven females [Mean ± SD: age = 20.8 ± 3.4 yr, height = 171.8 ± 10.0 cm, body mass = 70.4 ± 11.3 kg, body mass index (BMI; in kg.m^-2^) = 23.8 + 0.11]. Participants were assigned randomly to each experimental condition within a period of two weeks. Female participants were studied in days 1 to 7 of their menstrual cycle to minimise the influence of cyclical changes in female hormones.

All participants were screened prior to the start of the testing in order to determine that they are free from illness and any type of orthopaedic limitation or injury, and also that they meet the exclusion criteria defined as follows: A) History of any cardiovascular or respiratory disease, hypertension, liver or kidney disease, musculoskeletal or neuromuscular or neurological disease, autoimmune disease, cancer, peptic ulcers or anaemia. B) Taking medications, as well as a family history of heart problems, high blood pressure, and/or stroke, and being pregnant or breastfeeding. C) Consuming any ergogenic aid or above habitual caffeine consumption rate (200 mg/day) for at least one week prior to the study based on all types of caffeinated beverages (coffee, energy drinks, soft drinks, caffeine supplements or medications). All Participants refrained from taking any supplements for the duration of the study and were instructed to refrain from strenuous exercise or alcohol and caffeine consumption for at least 24 hours before each test. Participants have also completed a 24-hrs food diary and were asked to replicate it before the second visit.

### Experimental procedures and protocols

All participants reported to the Physiology Laboratory on two separate occasions following 10 hrs overnight fast, and each testing session (between 0700 and 1000) was separated by at least three days within two weeks period. Participants who had no previous laboratory experience were habituated in a separate laboratory visit, which included repeating the exercise and ingestion protocol within similar laboratory conditions. Anthropometric measurements included stature and body mass (Seca Alpha, Hamburg, Germany). During each visit participants ingested either 1000 mg (2× 500 mg capsule) YM or hydroxypropyl methylcellulose placebo empty capsules (PLC). Two capsules with similar coatings of either YM and PLC capsules were placed within an empty water cup and taken in the same way with a 100 ml of water. The YM capsules contained a standardised ground YM leaves (batch number 0422009/2012) with a natural content of approximately 1.5% caffeine (Rio Trading Company, Brighton, United Kingdom). Immediately following the ingestion, participants rested for 60 minutes in a semi-recumbent position in quiet laboratory condition. For the estimation of FAO and CHO at rest and during exercise, breath by breath cardiorespiratory measurements included oxygen uptake $\left(\dot{V}O_{2}\right)$, carbon dioxide production $\left(\dot{V}{\mathrm{CO}}_{2}\right)$ and respiratory exchange ratio (RER), using an online gas analyzer (Metalyzer Cortex 3B, Leipzig, Germany). The metabolic measurement system was calibrated prior to each test as follows: flow sensor and gas analyzers were calibrated using calibration gases of known concentration (16% for O_2_, and 5% for CO_2_), and for the gas volume a 3 liter volume calibration syringe (Hans Rudolph, Kansas, USA) was used.

### Exercise protocol

All participants followed an incremental exercise assessment using an electromagnetically braked cycling ergometer (Schoberer Rad Messtechnik, SRM, Ergo, Julich, Germany). The ergometer was calibrated before use and similar cycling positions were applied in both tests for each participant, which included adjusting the handlebar and saddle height and distance, crank length and toe clip positions during the first visit and re-applying the same position in the following visit. The cycling protocol consisted of three-minute incremental stages that were initiated at and increased by 0.5 W.kg^-1^ of body mass. Participants cycled at 60–70 rpm throughout the whole test until volitional exhaustion defined as meeting the at least two of $\left(\dot{V}O_{2\mathrm{peak}}\right)$ termination criteria: RER value > 1.1, heart rate within 10 beats.min^-1^ of age-predicted maximum heart-rate, or achieving levelling-off of $\dot{V}O_{2}$. Peak power (P_peak_) was calculated as the highest power output achieved during the last completed incremental stage, plus the fraction of time spent in any final non-completed stage multiplied by the power increment. Similar verbal encouragement was provided to all participants throughout the exercise tests.

All tests were followed by a sufficient cool down for at least 20 min, in which participants consumed at least 200 ml of water, and instructed to stay hydrated and consume at least 2 litres of water during the day of the test.

### Blood sampling

Capillary blood samples (5 μ*l*) were collected from the pin prick of the finger tip, at rest, during the last 30 s of each incremental exercise stage and immediately after exhaustion at min 1, 3 and 5. Blood samples were further analyzed for blood lactate using a portable analyzer (Lactate Pro LP1710, Arkray inc. Japan).

### Data processing and statistical analysis

$\dot{V}O_{2}$, $\dot{V}CO_{2}$ and RER were averaged for the last minute of each 5 min of the 60 min rest, and averaged for the last minute of each incremental stage. RER was calculated as $\left(\dot{V}CO_{2}/\dot{V}O_{2}\right)$. FAO and CHO were estimated using the stoichiometric indirect calorimetry equations assuming minimal protein contribution during exercise [17] (Eq. 1 and 2). Caloric equivalents were applied to estimate EE_FAO_ and EE_CHO_.

(1) $$\text{FAO}=1.695\times \dot{V}O_{2}-1.701\times \dot{V}CO_{2}$$

(2) $$\text{CHO}=4.585\times \dot{V}CO_{2}-3.226\times \dot{V}O_{2}$$

All data were described as mean and standard deviation. A mixed measure ANOVA (YM x power outputs) was applied to detect the effects of YM ingestion on RER and FAO, with YM supplement as within factors, and stage power increments as between factors, and Bonferroni post hoc test was applied to analyse the differences at each power output increment. Paired *t*-test was applied to detect the differences between YM and PLC at baseline and at maximal exercise performance. For all statistics SPSS V21 was used and the significance level was set at p < 0.05.

## Results

Following the 60 min rest after YM ingestion, no significant difference was found for either resting BLC (1.4 ± 0.32 vs. 1.5 ± 0.30 mmol.l^-1^) or resting RER (0.82 ± 0.08 vs. 0.81 ± 0.05) for PLC vs. YM respectively.

**Figure 1:**
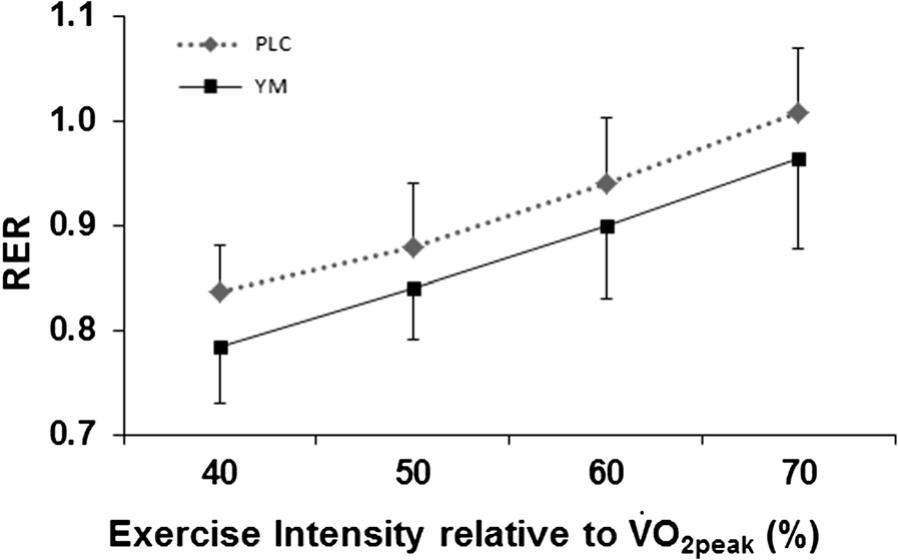
**Respiratory exchange ratio (RER) at submaximal intensities in YM compared with PLC *(P < *** **0.001, ANOVA main effects).**

**Figure 2:**
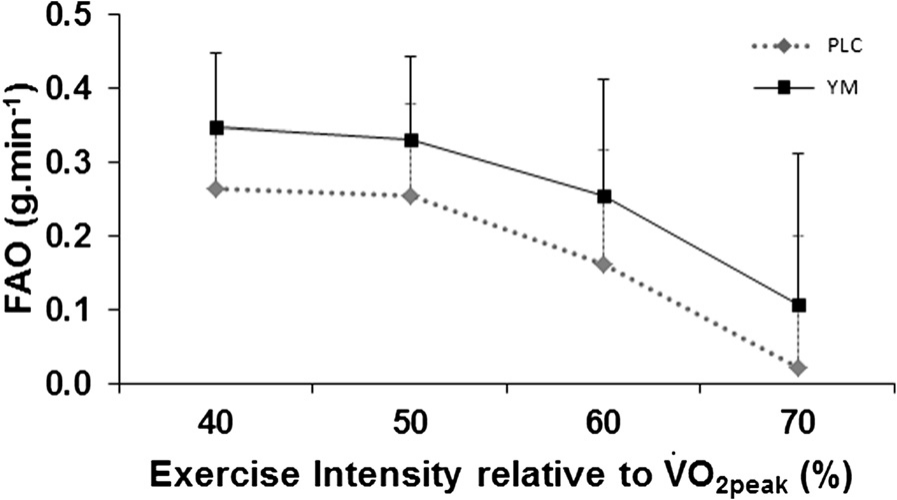
**Fat oxidation rate (FAO) at submaximal intensities for YM compared with placebo *(P < *** **0.001, ANOVA main effects).**

**Figure 3:**
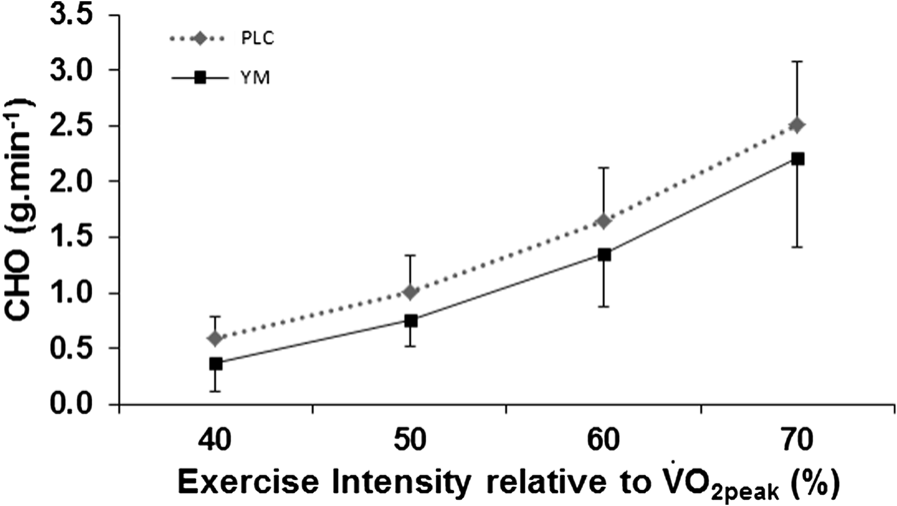
**Carbohydrate oxidation rate (CHO) at submaximal intensities for YM compared with placebo *(P < *** **0.001, ANOVA main effects).**

However, during exercise YM significantly reduced RER (*P* < 0.001, ANOVA main effects), (Figure 1) and increased FAO (*P* < 0.001, ANOVA main effects), (Figure 2). The YM effects on RER and on FAO were independent of the increase in power output (*P* < 0.001 in both conditions) and there was no interaction effects between the power output increase and YM supplement (Figures 1 and 2). Conversely, CHO was reduced in YM compared with PLC (Figure 3).

In terms of energy expenditure contribution, irrespectively of exercise intensity increase (no interaction effects) YM increased EE_FAO_ (*P* < 0.001), and decreased and EE_CHO_ (*P* < 0.01), (Table 1).

**Table 1:** **Energy expenditure contributions (Mean ± SD) from FAO (EE**_**FAO**_**) and CHO (EE**_**CHO**_**) at submaximal intensities in YM compared with PLC (all *P*** < **0.001, ANOVA main effects)**

**Exercise Intensity** $\left(\%\dot{V}O_{2\mathrm{peak}}\right)$	**EE**_**FAO-**_**PLC kJ. min**^**-1 **^**(kcal.min**^**-1**^**)**	**EE**_**FAO-**_**YM kJ. min**^**-1 **^**(kcal.min**^**-1**^**)**	**EE**_**CHO-**_**PLC kJ. min**^**-1 **^**(kcal.min**^**-1**^**)**	**EE**_**CHO-**_**YM kJ. min**^**-1 **^**(kcal.min**^**-1**^**)**
**40**	9.97 ± 3.27 (2.38 ± 0.78)	13.08 ± 3.73 (3.13 ± 0.90)	9.92 ± 3.27 (2.37 ± 0.78)	6.15 ± 4.19 (1.47 ± 1.00)
**50**	9.56 ± 4.70 (2.28 ± 1.12)	12.43 ± 4.22 (2.97 ± 1.00)	16.74 ± 5.57 (4.00 ± 1.33)	12.60 ± 4.06 (3.01 ± 0.97)
**60**	6.04 ± 5.82 (1.44 ± 1.39)	9.56 ± 5.92 (2.28 ± 1.41)	27.54 ± 7.95 (6.58 ± 1.90)	22.60 ± 8.00 (5.40 ± 1.91)
**70**	0.78 ± 6.68 (0.10 ± 1.00)	4.01 ± 7.69 (0.96 ± 1.83)	42.11 ± 9.33 (10.06 ± 2.23)	37.13 ± 13.48 (8.87 ± 3.22)

**Figure 4:**
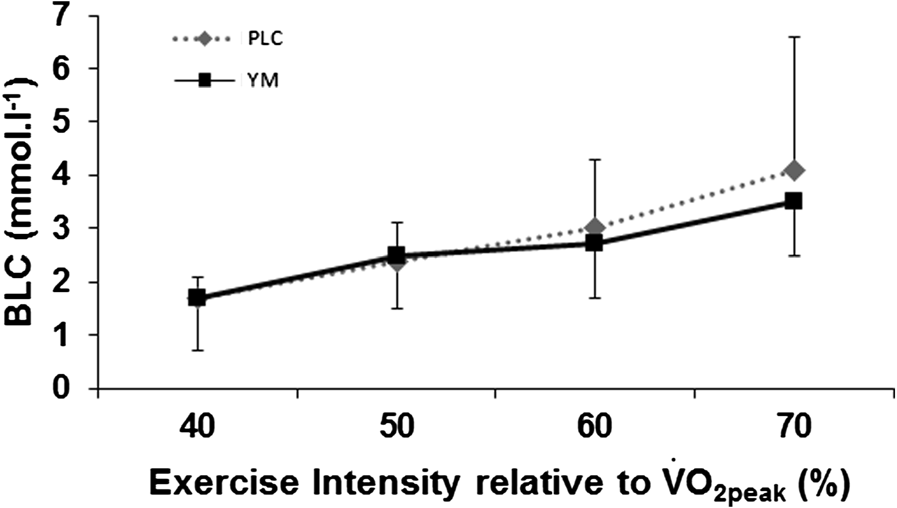
**Blood lactate concentration (BLC) at submaximal intensities for YM compared with placebo (*P =  *** **0.066, ANOVA main effects).**

There was also a trend, though not significant towards a reduced BLC (*P* = 0.066) in YM condition compared with PLC (Figure 4) at submaximal exercise. However, at peak level no difference was found in either peak BLC (BLC_peak_), $\dot{V}O_{2\mathrm{peak}}$, P_peak_ or peak RER (RER_peak_) in YM compared with PLC (Table 2).

**Table 2:** Maximal data (Mean ± SD) in YM compared with PLC

**P**_**peak **_**(W)**		$\dot{V}O_{2\mathrm{peak}}$ **(ml.kg**^**-1**^**.min**^**-1**^**)**		**BLC**_**peak **_**(mmol.l**^**-1**^**)**		**RER**_**peak**_	
**YM**	**PLC**	**YM**	**PLC**	**YM**	**PLC**	**YM**	**PLC**
222.3 ± 59.8	221.8 ± 66.8	38.8 ± 8.4	38.1 ± 8.7	9.5 ± 2.5	9.1 ± 2.7	1.14 ± 0.05	1.11 ± 0.07

## Discussion

The key finding of the present study is that YM ingestion augments FAO and reduces CHO reliance during exercise, over a wide range of exercise intensities, particularly in the light and moderate domains that are known to be effective training intensities and are often prescribed for a variety of population groups with the aim of weight loss, disease prevention and improving endurance exercise performance [13-15,18]. Augmented metabolic benefits of YM ingestion when combined with exercise could consequently contribute to the prevention and treatment of overweight and obesity associated metabolic health risks including diabetes and hyperinsulinaemia, hypercholestrolaemia and cancer [19,20].

In both treatment conditions FAO was increased similarly as a function of power output, but higher FAO was found in the YM condition at exercise intensities below 70% of $\dot{V}O_{2\mathrm{peak}}$ (Figures 1 and 2). Within this range of exercise intensities, FAO is well known to be utilised as a primary fuel source for energy expenditure (30-70%), while CHO predominates at heavy exercise intensities until reaching CHO saturation level (corresponding to RER = 1) [9,10,12]. Therefore, increased FAO and EE_FAO_ with YM ingestion in the light and moderate exercise intensity domains may augment both exercise dependent outcomes associated with those intensities [11,14], and may augment metabolic and anti-adiposity markers associated with YM ingestion, such as decreased differentiation of pre-adipocytes and reduced accumulation of lipids in adipocytes [3-5].

It is well documented that FAO and lipolysis increase several fold during exercise than at rest [10,21,22], and the present study is the first to demonstrate the positive effects of YM ingestion during exercise, particularly submaximal exercise, rather than at rest. Only one study reported a reduction in RER during 1–4 hrs of rest in healthy individuals who ingested 1 g of YM [8], which is similar to the present study. In comparison, the present study, using a similar dosage and following one hr rest, found that the differences in RER in YM vs. PLC become more prominent and significant during exercise (Figure 1), which corresponded to 24% increase in both FAO and EE derived from FAO (Table 1). This increase proposes YM as a promoter of fat metabolism during exercise.

The exercise induced metabolic effects found in the present study could be attributed to a number of reasons related to the major constituents of YM. Perhaps, the main effects YM function found on FAO are explained by central mechanisms and glycogen sparing of caffeine [23], which is found to have the highest concentration of 1% to 2% (naturally occurring ~1.5% in the present study) of dry weight of YM leaves and stem [24]. This caffeine amount (approximately 80 mg) is considered low compared with other multi-ingredient thermogenic supplements that reported increased FAO and EE (only at rest) where caffeine content exceeds 350 mg [25], which suggests that other active ingredients may have contributed to the present findings. The remaining reported compounds of YM include saponins, which are attributed to anti-lipolytic and hypocholestrolaemic properties when administered chronically, and caffeoyl derivatives (caffeic acid, chlorogenic acid, 3, 4-Dicaffeoylquinic acid, 4, 5-Dicaffeoylquinic acid and 3, 5-Dicaffeoylquinic acid) which are thought to have higher concentration compared with green or black tea and to have mainly anti-oxidant properties [1]. It has recently been shown that plasma lipid profile (triglycerides, fatty acids and total cholesterol) is ameliorated in mice fed with YM, combined with a positive effect on leptin’s central and peripheral induced feedback loop that regulates adipose tissues, energy balance and EE [26]. However, it is difficult to link these mechanisms with the acute effects during exercise in humans, though recent studies have suggested an acute effect of exercise on changes in lipid profile [27], which could be augmented by the ingestion of YM. It is also important to note that YM has a number of amino acids (i.e. Glutamic acid, Proline), minerals (P, Fe and Ca) and vitamins (C, B1 and B2) which have energy metabolism properties [1,3], which could influence exercise metabolic outcomes [28], though further research is required to determine their potential relationship.

Perhaps the closest supplement to compare the found increase in FAO contribution to total EE is green tea (Camella Sinesis). This is due to several similar active ingredients especially the phenolic antioxidant content in both YM and green tea, though catechin polyphenols have been shown to be highly abundant in green tea, while saponins and some caffeoyl derivatives are abundant in YM [1,3]. The present study administered 1 g of YM, almost half of the dosage of 1.8 g of green tea extract (no caffeine) administered by Venables *et al*. [14,16], and close to the dose of 525 mg of green tea extracts (375 mg catechins and 150 mg caffeine) with similar percentage of 1% of caffeine administered within 24 hrs by Dulloo *et al*. [19,29]. In comparison to the effects on EE reported in these studies, and only considering the similar active ingredients to those found in YM, the relatively small dosage of YM administered in the present study seem to be effective in further stimulating FAO and EE contribution to total FAO during exercise intensities in the moderate domain (Table 1), which is in agreement with the latter studies. However, the increase in FAO found in the present study of 0.09 g.min^-1^ is higher than a ~0.06 g.min^-1^ increase with green tea ingestion [16] and is close to 0.11 g.min^-1^ that was reported with an exercise training intervention [14]. Comparative analysis of YM and green tea that have used several measurement techniques have demonstrated higher content of active ingredients (approximately 50 more active ingredients) than those found in green tea [1,3], which suggest a potential important role for YM in metabolic health, and future research may elicit the potential health outcomes of combining YM with exercise training.

The study relied on an effective exercise protocol that is known reflect a wide range of exercise intensities, including effective training exercise intensities in the light and moderate domains (corresponding to RER ≤ 1) which are considered effective for a number of metabolic health, weight loss and cardiovascular risk reduction outcomes in a variety of population and age groups [14,30-32]. For example, training at intensities that correspond to maximal FAO has been associated with improved insulin sensitivity and fat loss [14]. Increased reliance on EE from FAO energy fuel sources with approximately 0.5-1.0 kcal.min^-1^ increase (*P* < 0.01) in EE from FAO and reduction in EE from CHO as induced by YM compared with PLC (Table 1) reflects a rightward shift in the intensity at the cross-over point defined as the power output when EE from CHO fuel sources predominates over EE from FAO, and hence it reflects an improvement in muscle glycogen sparing capacity, which is a known determining factor for endurance exercise performance [12]. Even though assessing YM effectiveness when combined with exercise training requires further investigations, the present study suggest a potential role for YM in increasing the training effectiveness at the cross-over point intensities [33,34], that have been tested within this study.

The YM dependent reduction (though non-significant) in BLC during exercise (Figure 4), is indicative of an effect on exercise tolerance and delaying fatigue mechanisms [35], are also in line with lower reliance on CHO as an energy fuel and increased reliance on FAO at exercise intensities corresponding to RER < 1 (Figure 3), which are all in the submaximal intensity domain, and agree with the dynamic interrelationship between BLC, CHO and FAO [11,12].

The present study relied on well-established metabolic markers that are based on capillary blood and cardiorespiratory gas measurements to demonstrate the YM effects during carefully selected exercise protocol with several intensity domains [11,15,18,34]. However, future studies need to further investigate the blood-based fat metabolic variables such as glycerol and non-esterified fatty acids, which would confirm the mechanisms behind any putative increase in lipid mobilisation and utilisation. Moreover, assessing both YM and plasma lipids post-ingestion for the active ingredients (eg. caffeic acid, chlorogenic acid, 3, 4-Dicaffeoylquinic acid, 4, 5-Dicaffeoylquinic acid and 3,5-Dicaffeoylquinic acid, phytosterols and saponins) would confirm the bioavailability of YM’s active ingredients.

It is also important to note that the YM effects on FAO and EE within the present protocol, particularly at sub-maximal intensities, may need to be further investigated using a variety of exercise protocols before any exercise training recommendations can be drawn. In particular, combining YM supplementation with supra-maximal and sprint type protocols, and training studies that utilises the recently publicised high-intensity interval training could be further investigated.

To conclude, acute ingestion of YM before exercise enhances fat metabolism during light and moderate exercise intensities, without negatively affecting maximal performance. These effects also suggest a glycogen sparing potential for exercise performance. Further research is required on specific long-term strategies that combine YM with exercise to accelerate weight loss outcomes and potentially enhance metabolic health outcomes.

## Abbreviations

YM: Yerba Maté; FAO: Fatty acid oxidation; CHO: Carbohydrate oxidation; EE: Energy expenditure; EE_FAO_: Energy expenditure derived from FAO; EE_CHO_: Energy derived from CHO; PLC: Placebo capsules; $\dot{V}O_{2}$: oxygen uptake; $\dot{V}CO_{2}$: carbon dioxide production; RER: Respiratory exchange ratio; $\dot{V}O_{{2\;}_{\mathrm{peak}}}$: peak oxygen uptake; BLC: Blood lactate concentration; BMI: Body Mass Index; BLC_peak_: peak blood lactate concentration; P_peak_: Peak power output; RER_peak_: peak respiratory exchange ratio

## Competing interest

The author declare that they have no competing interests.

## Author contributions

AA designed this study, analysed the data, prepared the figures, and prepared and finalized the manuscript.

## References

[B1] Heck CI de Mejia EG Yerba Mate Tea (Ilex paraguariensis): a comprehensive review on chemistry, health implications, and technological considerations J Food Sci 2007 72 9 R138 R151 10.1111/j.1750-3841.2007.00535.x 18034743

[B2] Bastos DHM De Oliveira DM Matsumoto RLT Carvalho PO Ribeiro ML Yerba maté: pharmacological properties, research and biotechnology Med Aromat Plant Sci Biotech 2007 1 37 46

[B3] Bracesco N Sanchez AG Contreras V Gugliucci A Recent advances on Ilex paraguariensis research: minireview J Ethnopharmacol 2011 136 3 378 384 10.1016/j.jep.2010.06.032 20599603

[B4] Borges MC Vinolo MA Nakajima K de Castro IA Bastos DH Borelli P Fock RA Tirapegui J Curi R Rogero MM The effect of mate tea (Ilex paraguariensis) on metabolic and inflammatory parameters in high-fat diet-fed Wistar rats Int J Food Sci Nutr 2013 64 5 561 569 10.3109/09637486.2012.759188 23317109

[B5] Kang YR Lee HY Kim JH Moon DI Seo MY Park SH Choi KH Kim CR Kim SH Oh JH Cho SW Kim SY Kim MG Chae SW Kim O Oh HG Anti-obesity and anti-diabetic effects of Yerba Mate (Ilex paraguariensis) in C57BL/6 J mice fed a high-fat diet Lab Anim Res 2012 28 1 23 29 10.5625/lar.2012.28.1.23 22474471 PMC3315195

[B6] Arçari DP Bartchewsky W Jr dos Santos TW Oliveira KA DeOliveira CC Gotardo ÉM Pedrazzoli J Jr Gambero A Ferraz LF Carvalho Pde O Ribeiro ML Anti-inflammatory effects of yerba maté extract (Ilex paraguariensis) ameliorate insulin resistance in mice with high fat diet-induced obesity Mol Cell Endocrinol 2011 335 2 110 115 10.1016/j.mce.2011.01.003 21238540

[B7] Arçari DP Bartchewsky W dos Santos TW Oliveira KA Funck A Pedrazzoli J de Souza MF Saad MJ Bastos DH Gambero A Carvalho Pde O Ribeiro ML Antiobesity effects of yerba maté extract (Ilex paraguariensis) in high-fat diet-induced obese mice Obesity (Silver Spring) 2009 17 12 2127 2133 10.1038/oby.2009.158 19444227

[B8] Martinet A Hostettmann K Schutz Y Thermogenic effects of commercially available plant preparations aimed at treating human obesity Phytomedicine 1999 6 4 231 238 10.1016/S0944-7113(99)80014-2 10589441

[B9] Romijn JA Coyle EF Sidossis LS Gastaldelli A Horowitz JF Endert E Wolfe RR Regulation of endogenous fat and carbohydrate metabolism in relation to exercise intensity and duration Am J Physiol 1993 265 3 Pt 1 E380 E391 8214047 10.1152/ajpendo.1993.265.3.E380

[B10] Van Loon LJ Greenhaff PL Constantin-Teodosiu D Saris WH Wagenmakers AJ The effects of increasing exercise intensity on muscle fuel utilisation in humans J Physiol 2001 536 295 304 10.1111/j.1469-7793.2001.00295.x 11579177 PMC2278845

[B11] Alkhatib A Duncan MJ, Lyons M Predictors of exercise performance Trends in Human Performance Research 2010 New York: NOVA Science Publisher 168 183

[B12] Brooks GA Mercier J Balance of carbohydrate and lipid utilization during exercise: the "crossover" concept J Appl Physiol 1994 76 6 2253 2261 7928844 10.1152/jappl.1994.76.6.2253

[B13] Pérez-Martin A Dumortier M Raynaud E Burn JF Fédou C Bringer J Mercier J Balance of substrate oxidation during submaximal exercise in lean and obese people Diabetes Metab 2001 27 4 Pt 1 466 474 11547220

[B14] Venables MC Jeukendrup AE Endurance training and obesity: effect on substrate metabolism and insulin sensitivity Med Sci Sports Exerc 2008 40 3 495 502 10.1249/MSS.0b013e31815f256f 18379212

[B15] Croci I Byrne NM Choquette S Hills AP Chachay VS Clouston AD O’Moore-Sullivan TM Macdonald GA Prins JB Hickman IJ Whole-body substrate metabolism is associated with disease severity in patients with non-alcoholic fatty liver disease Gut 2013 62 11 1625 1633 10.1136/gutjnl-2012-302789 23077135

[B16] Venables MC Hulston CJ Cox HR Jeukendrup AE Green tea extract ingestion, fat oxidation, and glucose tolerance in healthy humans Am J Clin Nutr 2009 87 3 778 784 18326618 10.1093/ajcn/87.3.778

[B17] Péronnet F Massicotte D Table of nonprotein respiratory quotient: an update Can J Sport Sci 1991 16 1 23 29 1645211

[B18] Achten J Gleeson M Jeukendrup AE Determination of the exercise intensity that elicits maximal fat oxidation Med Sci Sports Exerc 2002 34 1 92 97 10.1097/00005768-200201000-00015 11782653

[B19] Yusuf S Hawken S Ounpuu S Bautista L Franzosi MG Commerford P Lang CC Rumboldt Z Onen CL Lisheng L Tanomsup S Wangai P Jr Razak F Sharma AM Anand SS Obesity and the risk of myocardial infarction in 27,000 participants from 52 countries: a case–control study Lancet 2005 366 9497 1640 1649 10.1016/S0140-6736(05)67663-5 16271645

[B20] Blair SN Brodney S Effects of physical inactivity and obesity on morbidity and mortality: current evidence and research issues Med Sci Sports Exerc 1999 31 11 Suppl S646 S662 10593541 10.1097/00005768-199911001-00025

[B21] Arner P Kriegholm E Engfeldt P Bolinder J Adrenergic regulation of lipolysis in situ at rest and during exercise J Clin Invest 1990 85 893 898 10.1172/JCI114516 2312732 PMC296507

[B22] Wolfe RR Klein S Carraro F Weber JM Role of triglyceride-fatty acid cycle in controlling fat metabolism in humans during and after exercise Am J Physiol 1990 258 E382 E389 2106269 10.1152/ajpendo.1990.258.2.E382

[B23] Graham TE Caffeine and exercise: metabolism, endurance and performance Sports Med 2001 31 11 785 807 10.2165/00007256-200131110-00002 11583104

[B24] Ito E Crozier A Ashihara H Theophylline metabolism in higher plants Biochim Biophys Acta 1997 1336 2 323 330 10.1016/S0304-4165(97)00045-7 9305805

[B25] Outlaw J Wilborn C Smith A Urbina S Hayward S Foster C Wells S Wildman R Taylor L Effects of ingestion of a commercially available thermogenic dietary supplement on resting energy expenditure, mood state and cardiovascular measures J Int Soc Sports Nutr 2013 10 1 25 10.1186/1550-2783-10-25 23627832 PMC3651299

[B26] Hussein GM Matsuda H Nakamura S Hamao M Akiyama T Tamura K Yoshikawa M Mate tea (Ilex paraguariensis) promotes satiety and body weight lowering in mice: involvement of glucagon-like peptide-1 Biol Pharm Bull 2011 34 12 1849 1855 10.1248/bpb.34.1849 22130241

[B27] Shaheen HA Alpert PT Navalta J Tandy RD Young JC Santo AS The effect of acute endurance exercise on lipoproteins: a comparison of the nuclear magnetic resonance technique with the conventional lipid profile in healthy men Appl Physiol Nutr Metab 2014 39 2 233 237 10.1139/apnm-2013-0139 24476480

[B28] Kreider RB Wilborn CD Taylor L Campbell B Almada AL Collins R Cooke M Earnest CP Greenwood M Kalman DS Kerksick CM Kleiner SM Leutholtz B Lopez H Lowery LM Mendel R Smith A Spano M Wildman R Willoughby DS Ziegenfuss TN Antonio J ISSN exercise & sport nutrition review: research & recommendations J Int Soc Sports Nutr 2010 7 7 10.1186/1550-2783-7-720181066

[B29] Dulloo AG Duret C Rohrer D Girardier L Mensi N Fathi M Chantre P Vandermander J Efficacy of a green tea extract rich in catechin polyphenols and caffeine in increasing 24-h energy expenditure and fat oxidation in humans Am J Clin Nutr 1999 70 6 1040 1045 10584049 10.1093/ajcn/70.6.1040

[B30] Carnier J De Mello MT Ackel-DElia C Corgosinho FC Campos RM de Sanches PL Masquio DC Bueno CR Jr Ganen Ade P Martins AC Caranti DA Tock L Clemente AP Tufik S Dâmaso AR Aerobic training (AT) is more effective than aerobic plus resistance training (AT+RT) to improve anorexigenic/orexigenic factors in obese adolescents Appetite 2013 69 168 173 23764241 10.1016/j.appet.2013.05.018

[B31] Donnelly JE Honas JJ Smith BK Mayo MS Gibson CA Sullivan DK Lee J Herrmann SD Lambourne K Washburn RA Aerobic exercise alone results in clinically significant weight loss for men and women: midwest exercise trial 2 Obesity (Silver Spring) 2013 21 3 E219 E228 10.1002/oby.20145 23592678 PMC3630467

[B32] Alkhatib A Klonizakis M Effects of Exercise Training and Mediterranean Diet on Reducing Post-Menopausal Vascular Risk Clin Hemorheol Microcirc 2014 57 1 33 47 24004556 10.3233/CH-131770

[B33] Billat V Sirvent P Lepretre PM Koralsztein JP Training effect on performance, substrate balance and blood lactate concentration at maximal lactate steady state in master endurance-runners Pflugers Arch 2004 447 6 875 883 10.1007/s00424-003-1215-8 14740217

[B34] Venables MC Achten J Jeukendrup AE Determinants of fat oxidation during exercise in healthy men and women: a cross-sectional study J Appl Physiol 2005 98 1 160 167 15333616 10.1152/japplphysiol.00662.2003

[B35] Brooks GA Anaerobic threshold: review of the concept and directions for future research Med Sci Sports Exerc 1985 17 1 22 34 3884959

